# Maternal Characteristics, Intention, Self-Efficacy, Perceived Social Support, and Exclusive Breastfeeding Practice: Structural Equation Modeling Approaches

**DOI:** 10.3390/healthcare11010087

**Published:** 2022-12-28

**Authors:** Fang Li, Cailian Huang, Qian Lin, Yue Xi, Caihong Xiang, Cuiting Yong, Jing Deng

**Affiliations:** 1 Department of Pulmonary and Critical Care Medicine, The Second Xiangya Hospital, Central South University, Changsha 410011, China; lifang_csu@csu.edu.cn; 2 Hunan Centre for Evidence-Based Medicine, Changsha 410011, China; 3 Department of Epidemiology and Health Statistics, Xiangya School of Public Health, Central South University, Changsha 410078, China; 4 Maternal and Children Health Hospital of Changsha County, Changsha 410100, China; cailiang_huang@163.com; 5 Department of Nutrition Science and Food Hygiene, Xiangya School of Public Health, Central South University, Changsha 410078, China; linqian@csu.edu.cn (Q.L.); xiyue0404@csu.edu.cn (Y.X.); xch0622@csu.edu.cn (C.X.); yongcuiting@foxmail.com (C.Y.); 6 Hunan Provincial Key Laboratory of Clinical Epidemiology, Changsha 410078, China

**Keywords:** exclusive breastfeeding, Chinese mothers, influence factors, structural equation modeling

## Abstract

Breast milk is a perfect food for infants; however, the rate of exclusive breastfeeding is low. The relationship between exclusive breastfeeding practices and influencing factors is complex and remains unclear. This cross-sectional study was conducted in Changsha County, China, and 414 mothers were enrolled. An online questionnaire was used to collect data on general information, obstetrics and gynecology characteristics, the initial breastfeeding intention, breastfeeding practice, frequency of attending conventional breastfeeding programs before delivery, the status of breastfeeding self-efficacy, and the status of perceived social support. Structural equation modeling (SEM) was used to estimate the association between exclusive breastfeeding and potential risk factors of failing to practice exclusive breastfeeding for 6 months. The rate of exclusive breastfeeding for 6 months was 46.1%. The median and interquartile range of the scores for breastfeeding self-efficacy and perceived social support were 51.0 (18.0) and 68.0 (20.0), respectively. Factors that were statistically significant in the univariate analysis were included in the SEM and model fitness was acceptable based on the results. Exclusive breastfeeding for 6 months was directly associated with intention and self-efficacy, while it was indirectly associated with perceived social support and frequency of attending a breastfeeding program. The findings support the recommendation that comprehensive breastfeeding promotion strategies should be implemented to call on the intention and self-efficacy of breastfeeding mothers through various measures, such as education or providing medical and health services.

## 1. Introduction

Breast milk is the perfect food for infants and breastfeeding, especially exclusive breastfeeding, which provides infants with the best start in physical and mental development and lifelong health benefits [1,2]. Breastfeeding protects against illness and death in children and is beneficial for early childhood development. It decreases the risk of non-communicable diseases, such as childhood asthma, obesity, diabetes, and heart disease in later life [1,3]. On the other hand, breastfeeding promotes mothers’ well-being by improving birth spacing and reducing the risk of illness and disease, such as postpartum hemorrhage, breast cancer, and cardiovascular diseases [1,3]. In low- and middle-income countries (LMICs), increased breastfeeding could prevent 823,000 deaths in children under five annually, as well as 20,000 deaths due to breast cancer in mothers [3].

Despite the merits of breastfeeding, the rate of exclusive breastfeeding remains low. Indeed, only 37% of infants aged <6 months are exclusively breastfed in LMICs [3]. In China, exclusive breastfeeding rates were also unsatisfactory, ranging from 0.5% to 33.45% at the age of 6 months before 2019 [4,5,6,7,8,9]. This was far below the exclusive breastfeeding target of 50% at 6 months, which was set in the National Program of Action for Child Development in China (2011–2020) [10]. Thus, much effort is needed to scale up the exclusive breastfeeding rate in China.

According to the Cochrane Special Collections: Enabling breastfeeding for mothers and babies, many studies have discussed issues related to exclusive breastfeeding practice, including support for breastfeeding women, health promotion and enabling environments, caring for breastfeeding women and their babies, treatment of breastfeeding problems, and feeding practices for preterm babies/babies with additional needs and their mothers [11]. The factors associated with exclusive breastfeeding can be grouped into four major dimensions: infant, maternal, family, and social [4,6,7,11,12]. However, the results have been inconsistent. For example, Shi H. and colleagues conducted a national survey and reported that several factors were statistically associated with exclusive breastfeeding practices, including maternal age and maternal education [4]. However, Duan Y. et al. analyzed another nationally representative database and reported that the exclusive breastfeeding rate was not significantly associated with maternal age or educational level [7].

In addition, exclusive breastfeeding is influenced to more extent by multiple behavioral and psychological factors, such as intention, self-efficacy, and perceived social support [13,14,15]. Self-efficacy is considered to be related to exclusive breastfeeding; however, the results vary. Vakilian K. and colleagues reported a successful intervention program to improve the exclusive breastfeeding rate through home-based education on self-efficacy, while Monteiro J. and colleagues observed no association between breastfeeding self-efficacy and exclusive breastfeeding at 1 month postpartum [16,17].

Furthermore, previous studies showed that the above-mentioned influencing factors may affect each other [18,19]. Yang X. and colleagues reported that the intention to breastfeed, partner’s support, support from nurses/midwives, attending antenatal breastfeeding classes, time from childbirth to breastfeeding initiation, and previous breastfeeding experience were predictors of breastfeeding self-efficacy [20]. Kuswara K. and colleagues reported that breastfeeding intention, self-efficacy, and awareness of infant feeding guidelines were key factors associated with sustained exclusive breastfeeding for 4 months [21]. Thus, the intention to breastfeed and social support may be indirectly associated with exclusive breastfeeding via breastfeeding self-efficacy. This complex association can be illustrated by the structural equation modeling (SEM) approach, which is widely used to assess complex relationships and paths of health determinants [22,23,24].

Studies involving behavioral and psychological factors that influence exclusive breastfeeding were limited to Mainland China; two studies conducted among Chinese mothers outside Mainland China were noticed [21,25]. Given the discrepancy in family, social, and cultural backgrounds, the above-mentioned factors differed. Therefore, it is important to investigate the potential effects of these factors on exclusive breastfeeding. This will, in turn, provide healthcare providers with insights to seek interventions to increase the proportion of exclusive breastfeeding practices in Mainland China. In recent years, policies and actions aimed at promoting breastfeeding practices have been implemented in China. The China State Council introduced the National Program of Action for Child Development in China (2011–2020) in 2011, which set a goal of a 50% exclusive breastfeeding rate under 6 months. However, it is unclear whether, in the context of these policies, there is a direct or indirect association between exclusive breastfeeding practices and the aforementioned factors, such as breastfeeding self-efficacy, intention, and social support. With this background in mind, we conducted this study to (1) explore the factors influencing exclusive breastfeeding for 6 months and (2) discuss the mechanism among the influencing factors to increase the exclusive breastfeeding rate in Mainland China.

## 2. Materials and Methods

### 2.1. Ethics Approval

The study was approved by the Ethics Review Committee of the Xiangya School of Public Health, Central South University (XYGW-2021-036) and conducted in accordance with the guidelines of the Declaration of Helsinki. Trained researchers introduced this study to mothers. Informed consent was obtained from the participants by clicking on the confirmation button on the online questionnaire and participants were able to withdraw consent at any point during or after the survey, in which case the data would be deleted.

### 2.2. Study Design and Participants

This cross-sectional study was conducted at 23 primary medical and health institutions in Changsha County, Hunan Province, from January to February 2021. A total of 414 mothers of infants were enrolled in this study, with 15–20 mothers enrolled per primary medical and health institutions.

The inclusion criteria were (1) mothers who attended any one of these 23 primary medical and health institutions and (2) mothers who gave informed consent. The exclusion criteria were (1) mothers whose babies were younger than 6 months or older than 12 months, (2) mothers who could not practice breastfeeding due to medical concerns, (3) infants who were intolerant to breast milk, and (4) infants who had severe diseases, such as major malformations and genetic diseases.

The required sample size was 271, as calculated by PASS software (version 15.0 for Windows; NCSS LLC, Kaysville, UT, USA), and the prevalence of exclusive breastfeeding was 20.8% [26] with an allowable error of 10%. Considering the potential dropouts (20%), the dropout-inflated sample size was 339. Finally, a total of 414 participants were recruited for this study.

### 2.3. Data Collection

The outcome of interest was breastfeeding practice. An online questionnaire was developed by reviewing the literature and in consultation with experts. The questionnaire was then modified following a pilot survey. The resulting online questionnaire was used to collect the information indicated below.

#### 2.3.1. General Information, Obstetrics and Gynecology Characteristics, and Participation in the Breastfeeding Program

Collected general information included age, ethnicity, education, job, domicile, income, and marital status. Obstetric and gynecological characteristics included the number of children, history of gravidity, history of parturition, history of abortion, age at the last parturition, BMI before the last parturition, history of fetal or infant adverse pregnancy outcomes, delivery method of the last parturition, and history of maternal adverse pregnancy events during the last parturition. We also collected information on the frequency of attending a breastfeeding program.

#### 2.3.2. Breastfeeding Practice

Data on the initial intention and actual practice of breastfeeding were collected by using a self-reported questionnaire. The initial intention of breastfeeding was categorized into four levels: (1) artificial feeding, defined as feeding infants with food or liquids instead of breast milk; (2) mixed feeding, defined as feeding infants with other liquids or foods in addition to breast milk; (3) nearly exclusive breastfeeding, defined as feeding infants mainly with breast milk but providing a small amount of liquids, such as water and juice; and (4) exclusive breastfeeding, defined as feeding infants exclusively with breast milk without other foods or liquids, including water [27]. The actual practice of breastfeeding further was further grouped into two categories: whether or not exclusive breastfeeding was practiced for 6 months.

#### 2.3.3. Breastfeeding Self-Efficacy

The Chinese version of the Breastfeeding Self-Efficacy Scale of the Short Form was used to measure participants’ breastfeeding confidence [28,29]. The fourteen items were divided into two subscales: technique (items 1, 4, 5, 6, 8, 10, 11, 13, and 14) and intrapersonal thoughts (items 2, 3, 7, 9, and 12). All items are preceded by the phrase “I can always” and anchored with a 5-point Likert-type scale, where 1 indicates “not at all confident” and 5 indicates “always confident”.

#### 2.3.4. Perceived Social Support

The Chinese version of the Perceived Social Support Scale was used to measure the participants’ social support [30,31]. The 12 items of this scale were designed on a 7-point Likert-type scale ranging from 1 (very strongly disagree) to 7 (very strongly agree). Perceived adequacy of support from three sources was measured: family (items 3, 4, 8, and 11), friends (items 6, 7, 9, and 12), and significant others (items 1, 2, 5, and 10).

### 2.4. Statistical Analysis

Normally distributed continuous variables are presented as means and standard deviations, and otherwise by medians and interquartile ranges. The normality of the data was determined using the Kolmogorov–Smirnov test. Categorical variables are presented as numbers and proportions. Continuous variables were compared using one-way ANOVA or Mann–Whitney *U* tests; categorical variables were analyzed using chi-square tests or Fisher’s exact tests.

Structural equation modeling (SEM) was used to estimate the association between exclusive breastfeeding and potential risk factors of failing to practice exclusive breastfeeding for 6 months. Model fitness was determined using multiple indices including the ratio of the minimum discrepancy and degree of freedom (CMIN/DF), the goodness of fit index (GFI), the comparative fit index (CFI), the normed fit index (NFI), and the standardized root mean square residual (SRMR) [32]. SPSS Amos (version 21.0, IBM, New York, NY, USA) was used for SEM with the maximum likelihood estimation method.

All other statistical analyses were conducted using SPSS (version 25.0, IBM, New York, NY, USA). The significance level was set at *p* < 0.05.

## 3. Results

### 3.1. Social Economic Status, Obstetrics and Gynecology, History of Disease, and the Initial Breastfeeding Intention of Participants

A total of 414 participants were enrolled and 46.1% (191/414) of their babies were exclusively breastfed up until 6 months of age. The mean age of the mothers was 30.08 ± 4.41 years and the ethnicity of the vast majority of participants was Han. The mean age of the infants was 8.41 ± 2.32 months. A statistically significant difference in exclusive breastfeeding for 6 months was observed among mothers with different histories of fetal or infant adverse pregnancies, the initial intention of breastfeeding, and frequency of attending breastfeeding programs (all *p* < 0.05). The details are presented in Table 1 and Table 2, respectively.

### 3.2. Participants’ Breastfeeding Self-Efficacy

Table A1 shows participants’ breastfeeding self-efficacy. The scores for breastfeeding self-efficacy were 51.0 (18.0), 46.0 (19.0), and 56.0 (18.0) for all participants, participants who did not exclusively breastfeed for 6 months, and participants who exclusively breastfed for 6 months, respectively. Statistically significant differences were observed between the two groups (not exclusively breastfed vs. exclusively breastfed), including in the technique subscale score, intrapersonal thoughts subscale score, and total score of breastfeeding self-efficacy (all *p* < 0.001).

### 3.3. Perceived Social Support of the Participants

The perceived social support of participants is presented in Table A2. The median and interquartile range of the score of perceived social support was 68.0 (20.0). The scores of the three sources were 24.0 (8.0) for family, 22.0 (7.0) for friends, and 22.0 (7.0) for significant others. Statistically significant differences were observed between groups (not exclusively breastfed vs. exclusively breastfed, all *p* < 0.05), except for the subscore of significance for other support (*p* = 0.098).

### 3.4. Exclusive Breastfeeding for 6 Months in Structural Equation Modeling

**Figure 1:**
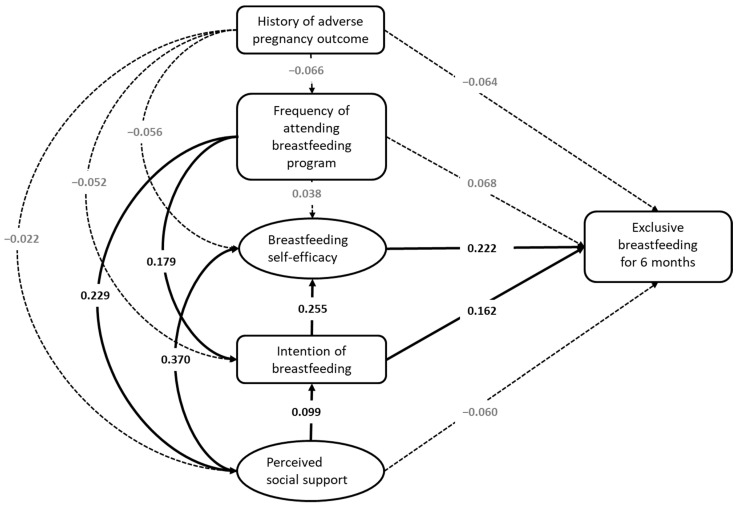
Structural equation model of exclusive breastfeeding at the age of 6 months (CMIN/DF = 5.451; GFI = 0.713; CFI = 0.866; NFI = 0.841; and SRMR = 0.0401). The coefficients of *p*-values higher than 0.05 are in gray and the corresponding paths are depicted with dotted lines. History of adverse pregnancy outcomes refers to adverse pregnancy outcomes relating to fetuses and infants, including embryo damage, extrauterine pregnancy, intrauterine growth retardation, birth defects, hydatidiform moles, neonatal death, low birth weight, premature delivery, macrosomia, and prolonged pregnancy.

Factors that were statistically significant in the previous analysis were included in the SEM, including a history of fetal or infant adverse pregnancy outcomes, frequency of attending a breastfeeding program, initial intention to breastfeed, the numerical total score of breastfeeding self-efficacy, and perceived social support (Figure 1). Table 3 lists the regression coefficients of the model. The fitness of the model was acceptable: CMIN/DF was 5.45, GFI was 0.71, CFI was 0.87, NFI was 0.84, and SRMR was 0.040.

Seven of the fifteen paths suggested in the hypothetical model were statistically significant (*p* < 0.05). The frequency of attending a breastfeeding program directly affected perceived social support and intention (*β* = 0.229, *p* = 0.004; *β* = 0.179, and *p* = 0.003, respectively) and indirectly affected intention, self-efficacy, and exclusive breastfeeding for 6 months (*β* = 0.023, *p* = 0.032; *β* = 0.136, *p* = 0.002; *β* = 0.058, and *p* = 0.002, respectively). Perceived social support directly affected intention and self-efficacy (*β* = 0.099, *p* = 0.042; *β* = 0.370, and *p* = 0.003, respectively) and indirectly affected self-efficacy and exclusive breastfeeding for 6 months (*β* = 0.025, *p* = 0.028; *β* = 0.104, and *p* = 0.002, respectively). Intention directly affected self-efficacy and exclusive breastfeeding for 6 months (*β* = 0.225, *p* = 0.002; *β* = 0.162, and *p* = 0.002, respectively) and indirectly affected exclusive breastfeeding for 6 months (*β* = 0.057 and *p* = 0.001). Self-efficacy directly affected exclusive breastfeeding for 6 months (*β* = 0.222 and *p* = 0.002). A history of fetal or infant adverse pregnancy outcomes indirectly affected exclusive breastfeeding for 6 months (*β* = −0.033 and *p* = 0.027).

## 4. Discussion

In this study, we found that the exclusive breastfeeding rate for 6 months was 46.1% in Changsha County and exclusive breastfeeding was directly associated with the initial intention to breastfeed and breastfeeding self-efficacy by SEM. The findings of this study may help increase exclusive breastfeeding practices in China.

Globally, the exclusive breastfeeding rate is approximately 37% and varies in different countries; the rate in high-income countries (HICs) is lower than the one in LMICs [11]. For example, less than 1% of babies are exclusively breastfed at 6 months in the UK in 2010, whereas approximately 20.7% of babies are exclusively breastfed at 6 months in China in 2013 [7,11]. The exclusive breastfeeding rate in our study was higher than that reported in previous studies conducted in China [4,5,6,7,8,9]. Duan Y. et al. reported the rate of exclusive breastfeeding under 6 months was 20.7% in a national cross-sectional survey conducted in 2013 [7]. Shi H. and colleagues reported a rate of 29.5% in a national cross-sectional survey conducted in 2018 [4]. A study conducted in Changsha reported a rate of 40% between 2013 and 2014 [33]. The discrepancy in the rate between this study and previous studies may be due to the recent advocacy initiative to promote breastfeeding in China. The China State Council introduced the “National Program of Action for Child Development in China (2011–2020)”, which set a goal of a 50% exclusive breastfeeding rate under 6 months [10]. With years of effort, the exclusive breastfeeding rate under 6 months has gradually increased; for example, between 2013 and 2018, it increased from 20.8% to 29.5% on a national level [4,26]. However, the exclusive breastfeeding rate was lower than the goal of 50% by 2020 [10]. A possible reason for this is the misunderstanding of mothers regarding exclusive breastfeeding. In this survey, nearly half of the participants (47.6%) thought that “exclusive breastfeeding refers to feeding infants with breast milk and ‘moderate water’” (data not shown). Mothers enrolled in this study did not realize that additional water was not allowed in exclusive breastfeeding practices. Thus, more work should be performed to correct misunderstandings and educate mothers on exclusive breastfeeding.

This study showed that initial intention to breastfeed was directly related to exclusive breastfeeding under 6 months, which supports the hypothesis that mothers’ strong breastfeeding intentions will lead to exclusive breastfeeding, which echoes the findings of previous studies [13,21,25,34]. Wu S. V. and colleagues observed a higher level of prenatal breastfeeding intention in the breastfeeding group than in the not breastfeeding group (9.80 ± 0.66 versus 8.63 ± 2.01, *p* = 0.001) [25]. Wilhelm S. L. and colleagues reported that women who intended to practice exclusive breastfeeding for 6 months were two times more likely to exclusively breastfeed for 6 months than those who did not [OR (95% CI): 2.19 (1.01–4.76)] [34]. These findings support the recommendation that healthcare professionals should call on the intentions of breastfeeding mothers.

The results showed that breastfeeding self-efficacy was also directly associated with exclusive breastfeeding practice under 6 months, which is consistent with previous studies [14,35,36,37,38]. A meta-analysis conducted by Brockway M. and colleagues examined the association between breastfeeding self-efficacy and exclusive breastfeeding by summarizing the studies with interventions on breastfeeding self-efficacy and the resultant exclusive breastfeeding rate [38]. They reported that for each 1-point increase in the mean breastfeeding self-efficacy score between the intervention and control groups, the odds of exclusive breastfeeding increased by 10% in the intervention group. Another meta-analysis observed that educational interventions using the breastfeeding self-efficacy theory were effective in improving the exclusive breastfeeding rate at postpartum 1~2 months [OR (95% CI): 1.69 (1.18–2.42)] [35].

The SEM showed that two factors, initial intention to breastfeed and breastfeeding self-efficacy, were directly associated with exclusive breastfeeding. However, other factors may be indirectly associated with exclusive breastfeeding via the above-mentioned two factors. For example, perceived social support may influence exclusive breastfeeding indirectly through its association with the initial intention of breastfeeding and breastfeeding self-efficacy. In addition, we observed a statistically significant association between the initial intention to breastfeed and self-efficacy. Previous studies have discussed such associations [18,19,20,39,40]. For example, Yang X. and colleagues conducted a cross-sectional study in China and found that breastfeeding self-efficacy in the immediate postpartum period could be predicted by the combination of intention to breastfeed, support from partners, support from nurses/midwives, attending antenatal breastfeeding classes, time from childbirth to breastfeeding initiation, and previous breastfeeding experience [20]. In contrast, in a multivariate logistic regression analysis, Mitra A. K. and colleagues reported that breastfeeding intention could be independently predicted by fewer children, past breastfeeding experience, breastfeeding knowledge, self-efficacy, and perceived social support [19]. Our study also found that the frequency of attending a breastfeeding program before delivery was indirectly and positively related to exclusive breastfeeding via initial intention to breastfeed. Thus, a comprehensive program targeting perceived social support, intentions, and self-efficacy of breastfeeding should be developed to increase exclusive breastfeeding rates. For example, the Action Plan for Breastfeeding Promotion (2021–2025) in China was launched in November 2021. This latest action was formulated to ensure the implementation of optimized fertility policies, safeguard the rights and interests of mothers and infants, and promote breastfeeding. The latest plan stipulates the following: 1. Disseminate scientific knowledge and vigorously conduct publicity and education on breastfeeding. 2. Improve the service chain and strive to strengthen breastfeeding consultation and guidance. 3. Improve policies and systems, and strive to build a supportive environment for breastfeeding. 4. Strengthen industry supervision and effectively crack down on illegal behaviors that endanger breastfeeding. This study provides evidence of the exclusive breastfeeding rate among postpartum women, as well as the direct and indirect relationship between exclusive breastfeeding practice, the initial intention of breastfeeding, breastfeeding self-efficacy, and other factors. Due to the limitations of the cross-sectional design and single-county sampling, the extrapolation of conclusions should be cautious. However, the main influencing factors found in this study correspond to the main tasks of China’s Action Plan for Promoting Breastfeeding (2021–2025). Therefore, our research not only provides effective evidence for the latest plan but also shows that the extrapolation of our research conclusions on influencing factors is acceptable. In addition, the result of our study can serve as the baseline data for Hunan Province, which is useful in evaluating the effect of China’s Action Plan for Promoting Breastfeeding (2021–2025). Second, due to the limited sample size, this study addresses only the influence of several maternal characteristics, initial intention, perceived social support, and breastfeeding knowledge. However, if research could be conducted with a larger sample, other potential factors such as infant characteristics, psychological condition, the influence of mass media, and previous breastfeeding experience could be included [12,41,42,43]. Third, data were collected via participant recall and using a self-reported questionnaire that may be subject to recall bias and social desirability bias.

## 5. Conclusions

The exclusive breastfeeding rate under 6 months in Changsha County was low. This study presents the direct relationship between exclusive breastfeeding practice and initial intention and self-efficacy of breastfeeding, as well as the indirect effect of social support and health education programs on exclusive breastfeeding practice. The findings support the recommendation that comprehensive breastfeeding promotion strategies should be implemented to call on the intention and self-efficacy of breastfeeding mothers through various measures, such as education or providing medical and health services.

## Figures and Tables

**Table 1:** The association between the demographic characteristic of mothers and exclusive breastfeeding for 6 months (*n* = 414).

Variable		Exclusive Breastfeeding				*p*-Value
		No		Yes		
		Case	%	Case	%	
Age	24 years old and below	22	9.9	26	13.6	0.227
	25–29 years old	65	29.1	61	31.9	
	30–34 years old	96	43.0	82	42.9	
	35 years old and above	40	17.9	22	11.5	
Ethnicity	Han (Majority)	212	95.1	181	94.8	0.889
	Minority	11	4.9	10	5.2	
Education	Primary school and below	18	8.1	24	12.6	0.436
	High school and college	116	52.0	89	46.6	
	Undergraduate	84	37.7	73	38.2	
	Graduate and above	5	2.2	5	2.6	
Job	Worker	60	26.9	37	19.4	0.407
	Administrative staff	12	5.4	13	6.8	
	Farmer	4	1.8	1	0.5	
	Commercial staff	9	4.0	11	5.8	
	Individual household	8	3.6	8	4.2	
	Teacher	11	4.9	13	6.8	
	Professional staff	18	8.1	23	12.0	
	Others ^a^	101	45.3	85	44.5	
Domicile	Local dweller	166	74.4	149	78.0	0.415
	Foreigner dwelling in Changsha for more than one year	51	22.9	40	20.9	
	Foreigner dwelling in Changsha for less than one year	6	2.7	2	1.0	
Family income per person (Yuan)	999 and below	7	3.1	6	3.1	0.846
	1000–2999	33	14.8	23	12.0	
	3000–4999	62	27.8	63	33.0	
	5000–6999	55	24.7	41	21.5	
	7000–9999	35	15.7	32	16.8	
	10,000 and above	31	13.9	26	13.6	
Marriage	Married	208	93.3	182	95.3	0.087 ^c^
	Married and separated	7	3.1	8	4.2	
	Others ^b^	8	3.6	1	0.5	

^a^ Including housewives; ^b^ Including women who were unmarried, divorced, or widowed; and ^c^ Tested by Fisher’s exact test.

**Table 2:** The association between characteristics of obstetrics and gynecology, history of disease, family history of disease and exclusive breastfeeding for 6 months (*n* = 414).

Variable		Exclusive Breastfeeding				*p*-Value
		No		Yes		
		Case	%	Case	%	
Number of children	1	107	48.0	94	49.2	0.802
	2 and above	116	52.0	97	50.8	
History of gravidity	1	69	30.9	63	33.0	0.163
	2	72	32.3	74	38.7	
	3 and above	82	36.8	54	28.3	
History of parturition	1	108	48.4	94	49.2	0.874
	2 and above	115	51.6	97	50.8	
History of abortion	0	113	50.7	107	56.0	0.434
	1	61	27.4	54	28.3	
	2	35	15.7	22	11.5	
	3 and above	14	6.3	8	4.2	
Age of the last parturition	24 years old and below	27	12.1	31	16.2	0.491
	25–29 years old	75	33.6	67	35.1	
	30–34 years old	90	40.4	73	38.2	
	35 years old and above	31	13.9	20	10.5	
BMI before the last parturition	Underweight	34	15.2	37	19.4	0.484
	Normal	155	69.5	120	62.8	
	Overweight	25	11.2	27	14.1	
	Obese	9	4.0	7	3.7	
History of fetal or infant adverse pregnancy outcomes ^b^	No	168	75.3	159	83.2	**0.049**
	Yes	55	24.7	32	16.8	
The delivery method of the last parturition	Spontaneous labor	126	56.5	113	59.2	0.511 ^a^
	Labor induced by drugs or obstetric apparatus	4	1.8	1	0.5	
	Cesarean	93	41.7	77	40.3	
History of maternal adverse pregnancy events during the last parturition ^c^	No	148	66.4	138	72.3	0.197
	Yes	75	33.6	53	27.7	
History of diseases during pregnancy	No	59	26.5	39	20.4	0.150
	Yes	164	73.5	152	79.6	
The initial intention of breastfeeding	Exclusive breastfeeding	124	55.6	144	75.4	**<0.001**
	Almost exclusive breastfeeding	39	17.5	29	15.2	
	Mixture feeding	53	23.8	18	9.4	
	Artificial feeding	7	3.1	0	0.0	
Frequency of attending a breastfeeding program	0	53	23.8	36	18.8	**0.026**
	1	46	20.6	33	17.3	
	2	45	20.2	30	15.7	
	3	22	9.9	14	7.3	
	4 and above	57	25.6	78	40.8	

^a^ Fisher’s exact test. ^b^ The fetal or infant adverse pregnancy outcomes include embryo damage, extrauterine pregnancy, intrauterine growth retardation, birth defects, hydatidiform mole, neonatal death, low birth weight, premature delivery, macrosomia, and prolonged pregnancy. ^c^ Maternal adverse pregnancy events included gestational diabetes mellitus, hypertensive disorders in pregnancy, hyperthyroidism during pregnancy, hypothyroidism during pregnancy, and hyperlipidemia during pregnancy.

**Table 3:** The direct, indirect, and total effects of the variables in the structural equation modeling.

Endogenous Variables		Exogenous Variables	*β*	SE	CR	*p*	SDE (*P*)	SIE (*p*)	STE (*p*)
Frequency of attending a breastfeeding program	<-	History of adverse pregnancy outcome	−0.252	0.188	−1.340	0.180	−0.066 (0.182)	-	−0.066 (0.182)
Perceived social support	<-	History of adverse pregnancy outcome	−0.057	0.128	−0.441	0.659	−0.022 (0.635)	-0.015 (0.166)	−0.037 (0.399)
Perceived social support	<-	Frequencies of a attending breastfeeding program	0.158	0.034	4.663	<0.001	0.229 (0.004)	-	0.229 (0.004)
Intention of breastfeeding	<-	History of adverse pregnancy outcome	−0.106	0.098	−1.084	0.278	−0.052 (0.289)	−0.015 (0.150)	−0.067 (0.193)
Intention of breastfeeding	<-	Perceived social support	0.077	0.039	1.979	0.048	0.099 (0.042)	-	0.099 (0.042)
Intention of breastfeeding	<-	Frequency of attending a breastfeeding program	0.096	0.026	3.639	<0.001	0.179 (0.003)	0.023 (0.032)	0.202 (0.003)
Breastfeeding self-efficacy	<-	Perceived social support	0.324	0.043	7.614	<0.001	0.370 (0.003)	0.025 (0.028)	0.395 (0.003)
Breastfeeding self-efficacy	<-	Frequency of attending a breastfeeding program	0.023	0.027	0.825	0.410	0.038 (0.372)	0.136 (0.002)	0.174 (0.002)
Breastfeeding self-efficacy	<-	History of adverse pregnancy outcome	−0.128	0.100	−1.271	0.204	−0.056 (0.215)	−0.033 (0.168)	−0.089 (0.071)
Breastfeeding self-efficacy	<-	Intention of breastfeeding	0.288	0.051	5.593	<0.001	0.255 (0.002)	-	0.255 (0.002)
Exclusive breastfeeding for 6 months	<-	Breastfeeding self-efficacy	0.118	0.029	4.025	<0.001	0.222 (0.002)	-	0.222 (0.002)
Exclusive breastfeeding for 6 months	<-	Perceived social support	−0.028	0.025	−1.127	0.260	−0.060 (0.320)	0.104 (0.002)	0.044 (0.346)
Exclusive breastfeeding for 6 months	<-	Intention of breastfeeding	0.097	0.030	3.246	0.001	0.162 (0.002)	0.057 (0.001)	0.218 (0.002)
Exclusive breastfeeding for 6 months	<-	Frequency of attending a breastfeeding program	0.022	0.016	1.406	0.160	0.068 (0.189)	0.058 (0.002)	0.126 (0.012)
Exclusive breastfeeding for 6 months	<-	History of adverse pregnancy outcome	−0.078	0.057	−1.366	0.172	−0.064 (0.169)	−0.033 (0.027)	−0.097 (0.048)

Abbreviations: *β*, regression coefficient; SE, standard error; CR, critical rate; *p*, *p*-value; SDE, standardized direct effect; SIE, standardized indirect effect; and STE, standardized total effect.

## Data Availability

The data presented in this study are available upon request from the corresponding author.

## Appendix A

**Table A1:** The association between breastfeeding self-efficacy and exclusive breastfeeding for 6 months (*n* = 414).

Variable	Exclusive Breastfeeding		*p*-Value
	No	Yes	
Technique subscale score	30.0 (11.0)	36.0 (10.0)	**<0.001**
Intrapersonal thoughts subscale score	16.0 (8.0)	20.0 (8.0)	**<0.001**
Total score of breastfeeding self-efficacy	46.0 (19.0)	56.0 (18.0)	**<0.001**

**Table A2:** The association between perceived social support and exclusive breastfeeding for 6 months (*n* = 414).

Variable	Exclusive Breastfeeding		*p*-Value
	No	Yes	
Family support score	23.0 (6.0)	24.0 (8.0)	**0.019**
Friends support score	22.0 (5.0)	24.0 (9.0)	**0.015**
Significant other support score	22.0 (6.0)	23.0 (9.0)	**0.098**
Total score of perceived social support	65.0 (16.0)	70.0 (24.0)	**0.025**
